# Burden and Determinants of Anemia in a Rural Population in South India: A Cross-Sectional Study

**DOI:** 10.1155/2018/7123976

**Published:** 2018-07-15

**Authors:** Matthew Little, Chloe Zivot, Sally Humphries, Warren Dodd, Kirit Patel, Cate Dewey

**Affiliations:** ^1^Department of Population Medicine, Ontario Veterinary College, University of Guelph, Guelph, ON, Canada; ^2^Department of Sociology and Anthropology, University of Guelph, Guelph, ON, Canada; ^3^School of Public Health and Health Systems, University of Waterloo, Waterloo, ON, Canada; ^4^International Development Studies, Menno Simons College, Canadian Mennonite University, Winnipeg, MB, Canada; ^5^Centre for Public Health and Zoonoses, Guelph, ON, Canada

## Abstract

**Background/Objectives:**

To determine the prevalence and determinants of blood haemoglobin level and mild, moderate, and severe anemia in a sample of adults from rural Tamil Nadu, India.

**Subjects/Methods:**

We recruited a sample of men and nonpregnant women aged 20 years and older. Clinical health measures included blood haemoglobin concentration and body mass index. We assessed associations between anemia outcomes and sociodemographic and dietary factors using linear and logistic regression modeling.

**Results:**

A total of 753 individuals (412 women and 341 men) participated in this study. The prevalence of anemia was 57.2% among women and 39.3% among men (*P*<0.001). Prevalence of anemia increased with age among men (*P*<0.001) but not women (*P*>0.05). Iron intake was low; 11.7% women and 24.1% of men reported iron intakes above recommended dietary allowances (*P*<0.001). Factors (OR (95% CI)) associated with mild or moderate anemia among women included television ownership (0.27 (0.13, 0.58)), livestock ownership (0.46 (0.28, 0.75)), refined grain consumption (1.32 (1.02, 1.72)), meat consumption (0.84 (0.71, 0.99)), and commercial agriculture production (mild: 4.6 (1.1, 18.8); moderate: 6.8 (1.98, 23.1)). Factors associated with mild, moderate, or severe anemia among men included rurality (0.50 (0.25, 0.99)), sugar consumption (1.04 (1.01, 1.06)), egg consumption (0.80 (0.65, 0.99)), and high caste (7.3 (1.02, 52.3)).

**Conclusion:**

Both women and men in this region may be particularly vulnerable to anemia, and future research must expand beyond dietary risk factors to examine the impacts of sociodemographic and environmental factors.

## 1. Introduction

Anemia is a condition characterized by a decreased number of red blood cells and has serious implications for the health, cognitive development, and productivity of adults and children worldwide. As of 2010, the global prevalence of anemia was approximately 32.9%, and this burden was borne primarily by women and children in low- and middle-income countries in Africa and south Asia [1]. Despite recent economic growth and prevention efforts, anemia remains particularly pervasive in India and is the largest cause of countrywide disability [2]. Data from the 2005-06 round of India's National Family Health Survey (NFHS) reveal that approximately 55% of women and 24% of men aged 15 to 49 suffer from anemia [3].

Previous research has identified several potential causes of anemia in the Indian context, such as low iron intake [4], limited vitamin C intake [5, 6], and lower gastric acidity relative to populations of European descent [7]. Among women, repeated childbearing, lactation [8], and poor access to nutritional supplements following menarche and during pregnancy may cause or further exacerbate anemia [9]. Furthermore, parasitic infections, such as hookworm and malaria, are also important causes of anemia [7, 9]. Such factors highlight the various sociocultural issues that influence anemia status, including poverty, micronutrient deficiencies, cultural and religious practices, access to health services, and poor awareness of the condition and preventive measures. Thus, the etiology of anemia in India is multifactorial and population-specific.

Most research on anemia in India has focused on urban settings [10, 11], pregnant women [12], and adolescents or children [12–14]. Considering anemia affects individuals of both sexes and all ages, there is a need for localized and context-specific studies to improve our knowledge of prevalence patterns and associated risk factors among general populations of adults, including nonpregnant women and men, in rural regions of India. This is particularly important in the southern state of Tamil Nadu, where anemia remains a severe public health problem across many districts [15] despite the existence of one of the most long-standing and robust social protection frameworks in the country, particularly with respect to food and nutrition schemes [16].

With this in mind, we undertook a cross-sectional study in a rural region of Tamil Nadu, South India, to determine prevalence estimates of mild, moderate, and severe anemia in adult men and nonpregnant women. We also assessed associations between anemia and various demographic, socioeconomic, and dietary risk factors. Overall, this study contributes to the body of literature describing and elucidating the burden and determinants of anemia in rural India for the purposes of informing policy, public health programming, and future research.

## 2. Methods

### 2.1. Ethics and Consent

Prior to the study, we obtained ethics approval from a Canadian university research ethics board. We were also granted permission for field research from the High Commission of India in Ottawa, Canada and from local authorities, including the* panchayat *council,* taluk* police supervisor, district police chief, district medical officer, local hospital staff, and the district Foreign Registration Office. Informed verbal consent was provided by all participants prior to enrollment in the study.

### 2.2. Sampling Frame and Sample Selection

We conducted a cross-sectional study in 17 villages in two rural* panchayats* in the Krishnagiri District of Tamil Nadu. Sampling methods have been published elsewhere [17]. Briefly, recruitment of individuals occurred through a randomized two-stage method, in which we approached a random sample of 8% of households in the sampling frame, and then employed the Kish method, developed by the World Health Organization (WHO) [18], to select a single household member (≥20 years of age) for the study. Self-reported pregnant women were excluded from the sampling frame. For each selected individual who agreed to participate, a follow-up appointment was scheduled. During the follow-up, descriptive information was collected on age, sex, education, income sources, and household possessions. Dietary intake was assessed with a validated food frequency questionnaire (FFQ), which was accompanied by a food atlas displaying photographs of dishes and serving sizes and collected information about the frequency of consumption and portion sizes of 223 dishes and foods [19]. The FFQ was paired with EpiNu® (Madras Diabetes Research Foundation, Chennai, IN), a database and software program that calculated overall intake of food categories and macro- and micronutrients based on frequency of consumption of dishes and laboratory-based nutritional analyses for each dish [20]. We assessed physical activity habits using self-reported estimates of “moderate” and “vigorous” activity in the realms of leisure, transportation, and work using the WHO's global physical activity questionnaire (GPAQ) [21]. The GPAQ was paired with locally relevant photographs depicting “moderate” and “vigorous” activities in all three realms.

### 2.3. Measurement and Definitions of Clinical, Socioeconomic, and Demographic Variables

Blood haemoglobin (Hb) concentration was assessed using capillary blood samples and a HemoCue® HB201+ analyzer (Hemocue AB, Angelholm, SE). Due to the high precision and repeatability of the HemoCue® analyzer [22], Hb concentration was measured only once for each participant. Mild anemia was defined as blood Hb concentration 110-129 g/L for men and 110-119 g/L for women. Moderate and severe anemia were defined as Hb concentration 80-109 g/L and <80 g/L respectively for men and women [23]. Stunting was defined as a height less than two standard deviations below the sex-specific reference Indian population median, calculated as <150.5 cm for men and <139.1 cm for women [24].

Socioeconomic status (SES) was assessed with an asset-based wealth index (hereafter referred to as wealth index) using a subset of 13 of 29 questions taken from the Standard of Living Index developed by the International Institute of Population Sciences (IIPS) for use in their National Family and Health Surveys (NFHS) [3]. Questions comprising the subset were selected for relevance to the study population. Attributes and possessions were weighted for a maximum score of 36 using weights developed by the IIPS based on* a priori* knowledge of their correlation with other measures of SES [25]. Caste data were categorized as low caste (comprised of scheduled castes and scheduled tribes), middle caste (comprised of other backwards castes and most backwards castes), and high caste (Brahmin caste). Binary variables were created for low caste and high caste, with middle caste as the referent. Religion was assessed as a binary variable (Muslim or Hindu). Migrant status was assessed as a binary variable (did or did not participate in temporary labour migration within the last 3 years).

A rurality index (RI) value was calculated for each individual, adapted from Weinert and Boik [26]. RI values were generated by summing the standardized values of (1) number of households in home village (assigned full weight) and (2) distance to primary health centre (in kilometers, assigned half weight). RI values were further standardized to a mean of zero and a SD of one. A higher RI value therefore represented a combination of a greater degree of isolation from the market village and other services and lower population density of household location.

### 2.4. Data Analysis

Data from the FFQ were processed with EpiNu®, which provided information on caloric consumption (kcal/day) and average daily macro- and micronutrient intake (g/day). Nutrient intake data were scaled to grams per 1000 kcal to account for differences in energy intake between participants. Iron intake values were compared to recommended dietary allowances published by the Indian Council of Medical Research [27]. Physical activity scores were calculated using the WHO's GPAQ Analysis Guide [21], which provided a total measure of Metabolic Equivalent (MET) minutes per week. Values were scaled to hours per day of moderate physical activity to improve ease of interpretation. Sedentary time was calculated as hours spent sitting per day and television time was calculated as hours spent watching television per day.

Statistical analyses were completed in STATA Version 13.0 (College Station, TX, USA). We calculated the crude prevalence of mild, moderate, and severe anemia. Prevalence estimates were then age- and sex-standardized using state-level age and sex data from the 2011 National Census [28]. We calculated sex-specific means and proportions for a number of baseline characteristics and explored differences between sexes using Student's *t*-tests (for means) and Pearson's chi-squared tests (for proportions). Following this, we assessed simple associations between a number of categorical sociodemographic and clinical characteristics and any-type (mild, moderate, or severe) anemia using unadjusted logistic regression analyses.

Using the regress command in STATA, we built two sex-specific multiple linear regression models with blood Hb concentration as the outcome. Following this, we used the mlogit command in STATA to build two sex-specific multinomial multivariable logistic regression with a four-level outcome: 0 = no anemia; 1 = mild anemia; 2 = moderate anemia; and 3 = severe anemia. For all models, we first assessed univariate correlates to identify associated factors at *P*-value<0.2. Following this, we included all identified factors in an initial multivariable model. We employed a backwards elimination model-building process in which we methodically eliminated nonsignificant variables at *P*-value<0.05, assuming no confounding if coefficients of remaining variables changed by less than 20% after removal of each variable. Multicollinearity was ruled out when post hoc diagnostics revealed variance inflation factor (VIF) values lower than 5 for each variable included in the final models [29]. For all models, we present below only those factors that were significantly associated with each outcome (*P*<0.05) following the backwards elimination process.

## 3. Results

Of the 812 individuals (397 men and 415 women) recruited for this study, 772 initially agreed to participate (96.0%), of whom 753 (93.5%) completed the FFQ and submitted blood samples. Overall response rates were 85.8% and 99.2% for men and women respectively (*P*<0.05). The primary reason for nonresponse was unavailability due to migrant labour work outside the home village; therefore, nonresponse bias was deemed negligible. Descriptive characteristics of participants by sex are displayed in Table 1.

Categorized clinical and sociodemographic characteristics of participants, as well as associations between characteristics and any-type (mild, moderate, or severe) anemia are displayed by sex in Table 2. Mean Hb concentrations were higher for men than women (*P*<0.001). Crude prevalences of mild, moderate, and severe anemia were 19.8%, 24.6%, and 2.9% respectively. Age- and sex-standardized prevalences of mild, moderate, and severe anemia were 19.9%, 22.6%, and 4.8%, respectively. Any-type anemia was almost twice as prevalent among women (57.2%) compared to men (35.2%) (*P*<0.001). Combined prevalence of moderate and severe anemia was three times higher among women (39.3%) than men (13.2%) (*P*<0.001). Prevalence was higher among men aged 60 years and older (53.8%) than those under the age of 60 (28.2%;* P*<0.01); however, prevalence was not significantly different between age groups among women. Mean (±standard deviation) iron intake was 13.4 mg/day (±12.77) among women and 14.2 (±13.47) mg/day among men, and only 11.7% of women and 24.1% of men reported iron intakes above recommended dietary allowances (*P*<0.001).

In all linear and logistic regression models, no confounding or multicollinearity was detected. Few factors were associated with blood Hb concentration among nonpregnant women in a multiple linear regression model (Table 3). An average iron intake above recommended dietary allowance cut-off was positively associated with Hb level. Ownership of a business was associated with increased blood Hb concentration. The adjusted R-squared value for this model was 0.027, indicating that 2.7% of the variance in the outcome variable was modeled by covariate patterns of exposure variables.

Several factors were associated with blood Hb concentration among men in a multiple linear regression model (Table 4). Increased age and participation in agriculture were negatively associated with blood Hb concentration. Fish consumption was associated with increased blood Hb concentration. Meanwhile, increased isolation from the market village was associated with increased blood Hb concentration. The adjusted R-squared value for this model was 0.21, indicating that 21% of the variance in the outcome variable was modeled by covariate patterns of exposure variables.

Several factors were associated with mild and moderate anemia among nonpregnant women in a multinomial multivariable logistic regression model (Table 5). Television ownership and livestock ownership were associated with lower odds of moderate anemia. Refined grain consumption was associated with increased odds of mild anemia. Meat consumption was associated with reduced odds of mild anemia. Individuals who were commercial agriculture producers had greater odds of having mild or moderate anemia.

Finally, five factors were associated with odds of mild, moderate, and/or severe anemia among men in a multinomial multivariable logistic regression analysis (Table 6). Increased age was associated with mild, moderate, and severe anemia. Increased rurality was associated with reduced odds of severe anemia. Sugar consumption was associated with increased odds of mild anemia, while egg consumption was associated with reduced odds of mild anemia. High caste was associated with increased odds of moderate anemia.

## 4. Discussion

We examined the burden of anemia in a sample of adult men and nonpregnant women in a rural region of Tamil Nadu, South India. Prevalence of anemia among women was consistent with the national rural average (57.4%) [3] but was lower than other regional populations in Tamil Nadu and elsewhere in India [30, 31]. The burdens of moderate and severe anemia among the sample population were considerably higher than national figures (15.0% and 1.8%, respectively), underscoring the severity of anemia as a public health concern in the study region [3]. While there is a dearth of prevalence data on anemia among men in India, our study found one of the highest recorded prevalence estimates in India, considerably higher than the national rural average (24%) [3] and other regional populations in South India [31, 32].

Mean blood Hb concentration was lower for women than men, a finding that is consistent with previous research across regions and ethnicities [33]. Women did not consume significantly more iron than men despite having increased requirements, and a greater proportion of women than men failed to meet sex-specific recommended dietary allowances for iron [27]. Older age was associated with lower Hb levels and increased odds of mild, moderate, and severe anemia among men. Indeed, prevalence among older men (≥60 years) was significantly higher than younger men. These findings correspond with existing research that suggests ageing is associated with a number of factors that may reduce blood Hb levels, including reduced haematopoietic stem cells, limited cell division, and reduced response to hormonal stimulation [34–36].

Iron deficiency anemia is a major concern in rural India and comprises up to 70% of all anemia cases, possibly due to the high prevalence of vegetarianism and limited access to iron supplements [4]. While the impact of diet on risk of anemia in rural India is debated [37], our results contribute to population-based [38] and clinical [39] evidence that suggests inadequate iron intake can lead to iron deficiency and anemia. In our study, consumption of iron above the recommended dietary allowance was associated with improved blood Hb status among women. We also found that meat consumption and egg consumption were associated with lower odds of moderate and mild anemia among women and men respectively, while fish consumption was associated with higher blood Hb concentration in men. Furthermore, livestock ownership was associated with reduced odds of moderate anemia among women. These findings suggest that meat and fish, which are excellent sources of highly bioavailable heme iron, may improve blood Hb concentrations [40]. Indeed, vegetarianism may account for the association between high caste and increased odds of moderate anemia in men, since high caste (Brahmin) individuals tend to avoid meat products [41]. Relatedly, consumption of refined grain (e.g., polished white rice and flour) and sugar, products that are low in iron and may replace animal products and vegetables in the diet, were associated with increased odds of mild anemia among women and men respectively. Our findings therefore provide further population-based evidence that contributes to the ongoing debate about the importance of iron intake in determining iron deficiency and anemia in rural India.

While research on the epidemiology of anemia in India is typically focused on biological and dietary risk factors [42], ours is one of the first studies to examine associations between anemia and a wide range of behavioural, socioeconomic, and geographic factors. We found that a number of nondietary variables were associated with blood Hb concentration and risk of anemia. For example, ownership of a business was positively associated with blood Hb concentration in women. This finding supports other studies showing an association between higher SES and decreased prevalence and severity of anemia [43], possibly due to improved access to a diverse diet, health care, and education. These results and others suggest that, while examining immediate dietary factors is important, we should not overlook the role of social and economic factors that may influence risk of anemia by altering dietary consumption or through other causal pathways.

Increased rurality was associated with increased blood Hb and decreased odds of severe anemia in men. These results challenge previous research by Kusumayati and Gross, who suggest that increased distance to urban centres has a negative impact on the nutritional status of communities in Indonesia [44]. Further, Jones and colleagues found no association between anemia and urbanicity as measured by nighttime light intensity in rural Andhra Pradesh, India [3]. One possible explanation for this discrepancy is that, in the study context, isolated households consume an iron-rich diet due to their dependence on subsistence agriculture and livestock [45]. Indeed, the cultivation of iron-rich coarse cereals (e.g., finger millet) and animal husbandry are common practices among subsistence and smallholder farmers northwestern Tamil Nadu, and households further removed from urban or peri-urban centres may be more likely to engage in subsistence agricultural activities than those close to the market village. This hypothesis corresponds with additional findings that participation in commercial agriculture, which may reduce the land dedicated to subsistence cropping and livestock, was associated with lower Hb concentration in men and increased odds of mild and moderate anemia in women.

This study has several limitations. Due to its cross-sectional nature, we were unable to determine causation. This was a challenge due to the potential inclusion of individuals with prediagnosed anemia who may have altered their dietary intake or behaviour following diagnosis. Further limitations may have impacted results or confounded regression models. Specifically, while the HemoCue® method is widely accepted in clinical and epidemiological settings [22, 46–48], some validation studies indicate it may overestimate Hb levels [49]. Smoking can increase Hb levels, which may lead to confounding in regression models [50], although we saw no evidence of this in the analysis. Additionally, although the FFQ is considered a valid form of dietary assessment and has been used in both urban and rural studies in Tamil Nadu [17, 19], the limitations of FFQs are well-documented and include a tendency to overestimate food intakes and a susceptibility to social desirability and social approval biases [51]. Finally, there are additional factors that were not accounted for in our survey but may impact anemia. Such factors include physiological processes such as menstruation, lactation, parity, and menopause. The exclusion of such factors, which are largely sex-specific, may account for the lower adjusted R-squared value in the female linear regression model. Additional excluded factors include malaria, schistosomiasis, HIV, and soil transmitted helminths (e.g., hookworm infections) [15]. These diseases have the potential to significantly impair iron absorption or induce blood loss [52] in both sexes.

### 4.1. Conclusion

Our study contributes to the growing body of research documenting the high prevalence of anemia in southern India, while also highlighting associated risk factors in a region of rural Tamil Nadu. Our findings confirm and elucidate the multidimensional etiology of anemia in India. The implications of the research are considerable. Results of this study support previous findings that certain demographics (such as women and older men) are at higher risk of anemia in the Indian context. Further, our research highlights the complexity of associations between rurality, migration, occupation, agriculture, and SES and risk of anemia. The results of our research, which may contribute to a shift of discourse towards a multidimensional focus on socio-cultural, economic, and environmental factors, have important implications for policy design and public health. These results underscore the importance of a balanced and multifaceted policy approach to addressing anemia in rural India, including (1) subsidization and distribution of diverse and nutritious foods through food security schemes (e.g., the Public Distribution System) [53]; (2) targeting the most vulnerable populations (e.g., women, older populations, and those of lower SES) in food and social welfare programs; (3) developing or maintaining policies and programs directed towards reducing inequities in education, income, and access to services. Finally, while both research on and treatment for anemia should remain context-specific and individualized, interventions that address both age- and gender-specific iron intake and nondietary factors such as comorbid diseases, gender inequality, and socioeconomic processes should be further investigated.

## Figures and Tables

**Table 1:** Baseline characteristics of participants by sex for a sample of adults from rural Tamil Nadu, India.

Characteristic	Men (n = 341) % or mean ± SD	Women (n = 412) % or mean ± SD	Total (n = 753) % or mean ± SD
Age (years)	48.1 ± 14.77	46.3 ± 14.61	47.1 ± 14.70

Literate (%)	49.0	23.1	34.8

Family size (members in household)	4.5 ± 2.1	4.3 ± 2.0	4.4 ± 2.1

Physical activity (h/day moderate activity)	4.5 ± 3.80	3.6 ± 3.49	4.0 ± 3.66

Body mass index (kg/m^2^)	21.5 ± 3.91	22.0 ± 4.49	21.8 ± 4.24

Haemoglobin (g/L)	13.4 ± 2.48	11.5 ± 5.77	12.4 ± 4.66

Tobacco use (%) (smoking and/or *paan*)	48.7	31.1	39.0

Current alcohol consumer (%)	50.0	38.0	44.0

Dietary consumption			
Total energy (kcal/day)	2583 ± 800.0	2219 ± 629.4	2384 ± 734.1
Alcohol (g/day)	19 ± 61	1 ± 4.3	9 ± 42.1
Carbohydrates (g/1000 kcal)	178 ± 16.2	180 ± 12.8	180 ± 14.5
Fat (g/1000 kcal)	20 ± 5.2	20 ± 5.4	20 ± 5.3
Dietary fibre (g/1000 kcal)	22 ± 5.3	22 ± 4.9	22 ± 5.1
Dairy products (g/1000 kcal)	92 ± 75.0	71± 65.6	81 ± 68.0
Pulses and legumes (g/1000 kcal)	27 ± 11.7	28 ± 11.6	27 ± 11.7
Egg, fish, and poultry (g/1000 kcal)	3 ± 5.5	3 ± 4.9	3 ± 5.2
Red meat (g/1000 kcal)	0.9 ± 0.76	0.9 ± 0.70	0.9 ± 0.73
Fruits (g/1000 kcal)	56 ± 42.1	63 ± 49.8	60 ± 46.6
Leafy vegetables (g/1000 kcal)	6 ± 6.4	6 ± 7.5	6 ± 7.0
Other vegetables (g/1000 kcal)	10 ± 8.6	11 ± 9.5	10 ± 9.1
Iron intake (mg/day)	14 ± 6.7	13 ± 6.6	14 ± 6.6

**Table 2:** Clinical and sociodemographic characteristics and associations with any-type anemia (mild, moderate, or severe) by sex for a sample of adults from rural Tamil Nadu, India.

Characteristic	Men (n = 341)		Women (n = 412)	
	n (%)	Crude OR, any-type anemia (95% CI)	n (%)	Crude OR, any-type anemia (95% CI)
Anemia status (Hb concentration)				
Healthy (men/women: ≥130/≥120 g/L)	221 (64.8)	Ref	176 (42.7)	Ref
Mild anemia (men/women: 110-129/110-119 g/L)	75 (22.0)	NA	74 (18.0)	NA
Moderate anemia (80-109 g/L)	37(10.9)	NA	148 (35.9)	NA
Severe anemia (<80 g/L)	8 (2.3)	NA	14 (3.4)	NA

Family size				
≤2	42 (12.3)	Ref	83 (20.1)	Ref
3-5	214 (62.8)	0.50 (0.3, 1.0)*∗∗*	239 (58.0)	0.7 (0.4, 1.2)
>5	85 (24.9)	0.40 (0.2, 0.9)*∗∗*	90 (21.8)	0.9 (0.5, 1.7)

Age categories				
20-35	84 (24.6)	Ref	125 (30.3)	Ref
36-60	186 (54.5)	3.0 (1.5, 5.8)*∗∗∗*	220 (53.4)	1.08 (0.7, 1.7)
>60	71.0 (20.8)	8.4 (3.9, 18.0)*∗∗∗*	67 (16.3)	1.03 (0.6, 1.9)

BMI categories (kg/m^2^)				
Normal weight (≥18.5 & <23)	139 (40.8)	Ref	162 (39.3)	Ref
Underweight (<18.5)	84 (24.6)	2.3 (1.3, 4.1)*∗∗∗*	85 (20.6)	1.1 (0.6, 1.8)
Overweight (≥23 & <25)	56 (16.4)	1.9 (1.0, 3.8)*∗∗*	62 (15.0)	0.9 (0.5, 1.7)
Obese (≥25)	62 (18.2)	1.4 (0.7, 2.7)	103 (25.0)	1.1 (0.7, 1.8)

Stunted (height for men/women: <150.5 cm/<139.1 cm)	14 (4.1)	1.6 (1.0, 2.5)	6 (1.5)	1.4 (0.3, 7.7)

Tobacco use (*paan *and/or smoking)	169 (49.6)	1.1 (0.7, 1.7)	128 (31.1)	0.5 (0.4, 0.7)

Education				
Illiterate	174 (51.0)	Ref	315 (76.5)	Ref
Literate but did not complete primary school	94 (27.6)	0.7 (0.4, 1.3)	48 (11.7)	0.9 (0.5, 1.6)
Finished primary school	59 (17.3)	0.5 (0.3, 1.0)*∗∗*	46 (11.2)	0.7 (0.4, 1.3)*∗*
Finished secondary school	14 (4.1)	1.3 (0.4, 4.0)	3 (0.7)	1.5 (1.2, 1.9)

Primary occupation^†^				
Subsistence/small-scale agriculture producer or labourer	211 (61.9)	Ref	314 (76.2)	Ref
Commercial agriculture producer	48 (14.1)	1.0 (0.5, 1.9)	27 (6.6)	0.5 (0.2, 1.1)*∗*
Entrepreneur, business employee, or civil servant	39 (11.4)	0.8 (0.4, 1.6)	25 (6.1)	1.7 (0.69, 4.2)
MGNREGA^‡^	4 (1.2)	3.2 (0.3, 35.8)	14 (3.4)	1.8 (0.5, 5.7)
Migrant labour	4 (11.7)	0.40 (0.2, 0.9)*∗∗*	32 (7.8)	0.9 (0.4, 1.9)

Wealth index (by tertile)				
Low	108 (31.7)	Ref	200 (48.5)	Ref
Middle	112 (32.8)	0.7 (0.4, 1.2)	121 (29.4)	0.5 (0.3, 0.9)*∗∗*
High	121 (35.5)	0.6 (0.4, 1.1)*∗*	91 (22.1)	0.8 (0.5, 1.3)

Caste				
Low	43 (12.6)	Ref	52 (12.6)	Ref
Middle	271 (79.4)	1.7 (0.8, 3.5)*∗*	315 (76.4)	0.8 (0.5, 1.5)
High	27 (7.9)	1.8 (1.1, 2.5)*∗∗*	45 (10.9)	1.4 (0.6, 3.3)

Religion				
Hindu	327 (95.9)	Ref	386 (9.4)	Ref
Christian	4 (1.2)	0.6 (0.1, 5.8)	8 (1.9)	2.0 (0.4, 10.4)
Muslim	10 (2.9)	1.2 (0.3, 4.3)	18 (4.4)	0.4 (0.2, 1.2)

NA: not applicable, predictor perfectly predicts outcome; Ref: referent category.

*∗P*<0.2; *∗∗P*<0.05; *∗∗∗P*<0.01  .

^†^Primary occupation defined as the income- or livelihood-generating activity that takes up most amount of time.

^‡^Mahatma Gandhi Rural Employment Guarantee Act Scheme.

**Table 3:** Factors associated with blood haemoglobin concentration (g/dL) in a multiple linear regression model for a sample of non-pregnant women (>19 years) in rural southern India.

Variable	*β* Coefficient	*P*-value
Local business owner (Y/N)	2.10	0.019

Iron intake above recommended dietary allowance (Y/N)	1.9	0.036

Constant	15.1	<0.001

**Table 4:** Factors associated with blood haemoglobin concentration (g/dL) in a multiple linear regression model for a sample of men (>19 years) in rural southern India.

Variable	*β* Coefficient	*P*-value
Age (years)	-0.064	<0.001

Distance to market town (km)	0.052	0.006

Commercial agriculture producer (Y/N)	-1.02	0.016

Fish consumption (g/1000 kcal)	0.41	0.001

Stunting (Y/N)	-1.37	0.025

Constant	16.1	<0.001

**Table 5:** Factors associated with mild, moderate, and/or severe anemia in a multinomial logistic regression model^†^ for a sample of 412 nonpregnant women (>19 years) in rural southern India.

Variable	Mild anemia OR (95% CI)	Moderate anemia OR (95% CI)	Severe anemia OR (95% CI)
Television ownership (Y/N)	0.52 (0.19, 1.45)	0.27 (0.13, 0.58)*∗∗∗*	0.89 (0.02, 2.32)

Livestock ownership (Y/N)	0.61 (0.33, 1.11)*∗*	0.46 (0.28, 0.75)*∗∗*	0.76 (0.24, 2.39)

Refined grain consumption (g/1000 kcal)	1.01 (1.00, 1.02)*∗∗*	1.00 (0.99, 1.01)	1.00 (0.98, 1.02)

Meat consumption (g/1000 kcal)	0.88 (0.73, 1.06)*∗*	0.84 (0.71, 0.99)*∗∗*	0.49 (0.19, 1.30)*∗*

Commercial agriculture producer	4.6 (1.1, 18.8)*∗∗*	6.8 (1.98, 23.1)*∗∗∗*	4.0 (0.39, 41.4)

^†^No anemia as referent; dependent variable is a four-level outcome: 0 = no anemia; 1 = mild anemia; 2 = moderate anemia; 3 = severe anemia.

*∗P*<0.2; *∗∗P*<0.05; *∗∗∗P*<0.01.

Mild anemia, <120 g/dL; moderate anemia, 110-119 g/dL; severe anemia, 70-109 g/dL.

**Table 6:** Factors associated with mild, moderate, and/or severe anemia^†^ in a multinomial logistic regression model^†^ for a sample of 341 men (>19 years) in rural southern India.

Variable	Mild anemia OR (95% CI)	Moderate anemia OR (95% CI)	Severe anemia OR (95% CI)
Age (years)	1.04 (1.01, 1.06)*∗∗∗*	1.05 (1.02, 1.09)*∗∗∗*	1.1 (1.03, 1.18)*∗∗∗*

Rurality index	0.95 (0.65, 1.41)	0.97 (0.60, 1.57)	0.50 (0.25, 0.99)*∗∗*

Sugar consumption (g/1000 kcal)	1.04 (1.01, 1.06)*∗∗*	1.01 (0.98, 1.05)	1.01 (0.94, 1.09)

Egg consumption (g/1000 kcal)	0.80 (0.65, 0.99)*∗∗*	0.88 (0.58, 1.34)*∗*	0.88 (0.58, 1.34)

High caste (Y/N)	3.2 (0.48, 21.5)	7.3 (1.02, 52.3)*∗∗*	4.5 (0.65, 32.5)

^†^No anemia as referent; dependent variable is a four-level outcome: 0 = no anemia; 1 = mild anemia; 2 = moderate anemia; 3 = severe anemia.

Mild anemia, Hb<130 g/dL; moderate anemia, Hb 110-129 g/dL; severe anemia, 80-109 g/dL.

*∗P*<0.2; *∗P*<0.05, *∗∗P*<0.01.

## Data Availability

The data used to support the findings of this study have not been made available as per the confidentiality agreement presented to study participants during the consent process.
